# Machine learning approaches for improvement of patient safety in surgery

**DOI:** 10.1186/s13037-024-00422-y

**Published:** 2024-12-20

**Authors:** Philip F. Stahel, Kathryn Holland, Roy Nanz

**Affiliations:** 1 Mission Health, 509 Biltmore Ave., Asheville, NC 28801 USA; 2 Department of Surgery, East Carolina University, Brody School of Medicine, Greenville, NC USA; 3 Department of Specialty Medicine, Rocky Vista University, College of Osteopathic Medicine, Parker, CO USA

**Keywords:** Artificial intelligence, Machine learning, Predictive analytics, Patient safety in surgery

Traditional patient safety protocols are based on the historic principle of the Hippocratic oath intended to abstain from inflicting harm on our patients (*”Primum non nocere”*) [1]. In spite of this noble vision, patients frequently remain caught in the ‘friendly fire’ of preventable surgical complications and hospital-acquired adverse conditions, which are widely regarded as an unfortunate side-effect of modern healthcare [2]. Ironically, the plethora of stringent regulatory compliance-mandated protocols and globally disseminated patient safety checklists still fail to protect our patients from medical errors in the modern age of patient safety today [3]. Our continued quest towards “Goal Zero“ for preventable harm requires a pragmatic reassessment of the historically failed approaches in the patient safety arena [4, 5]. It is time for a paradigm shift from the traditional approach of counting and analyzing medical errors, with the intent of preventing similar occurrences to harm a different patient in the future, to effectively predicting and preventing an adverse event before harm reaches the patient [6]. The new age of artificial intelligence allows for novel machine learning approaches to support predictive analytics tools which can identify a patient at risk of sustaining harm before the adverse event occurs [7]. The latest special collection in *Patient Safety in Surgery* is dedicated to covering a variety of aspects pertinent to machine learning approaches for the improvement of surgical patient safety (www.biomedcentral.com/collections/MLPS).

A total of eight peer-reviewed articles were published in the collection from 2022 to 2024. A study from São Paulo University in Brazil [8] investigated the hypothesis that an AI approach would improve the quality and sensitivity of prostate cancer detection and grading in 6,982 image sections from 32 radical prostatectomy specimens (Fig. 1). The authors found that a “deep learning“ approach for AI-supported analysis showed a high sensitivity and specificity for detection and grading of cancer stages beyond the baseline inter-pathologist variance in the the interpretation of human prostate cancer specimens [8]. A different study from Bar-llan University in Israel assessed the value of machine learning for predicting risk factors which contribute to “never events“ in the operating, including wrong-site surgery and unintentionally retained foreign objects [9]. These results demonstrated impressively that selected variables had an impact of > 900% on predicting the occurrence of a “never event“ by machine learning analysis, including discrepancies in the second and third count of surgical instruments and sponges [9]. Conversely, standard elements in the surgical safety checklist appeared to have minimal impact or no impact at all on adverse event prediction (Fig. 2). The third paper in the collection represents a retrospective propensity-matched cohort study from a tertiary referral academic medical center in the United States [10]. The authors investigated the “Rothman Index“ (RI) as a predictive analytics tool to predict and prevent unsafe patient downgrades with subsequent unplanned readmissions to the intensive care unit (ICU) [10]. The RI represents a real-time composite measure of medical acuity in hospitalized patients and provides a predictive analytics model for continuous monitoring of a patient’s clinical status and improvement or deterioration over time (Fig. 3). During a 12-months study time-window, a total of 5,261 ICU patients were included in analysis, of which 212 patients (4.0%) had an unplanned readmission to the ICU within 7 days [10]. The results from this study identified the RI as a sensitive predictor of unanticipated readmissions to ICU which were associated with significantly increased mortality and hospital length of stay. The authors therefore concluded that the RI should be considered as a real-time objective measure for prediction of a safe downgrade from ICU to a lower level of care [10].

**Fig. 1:**
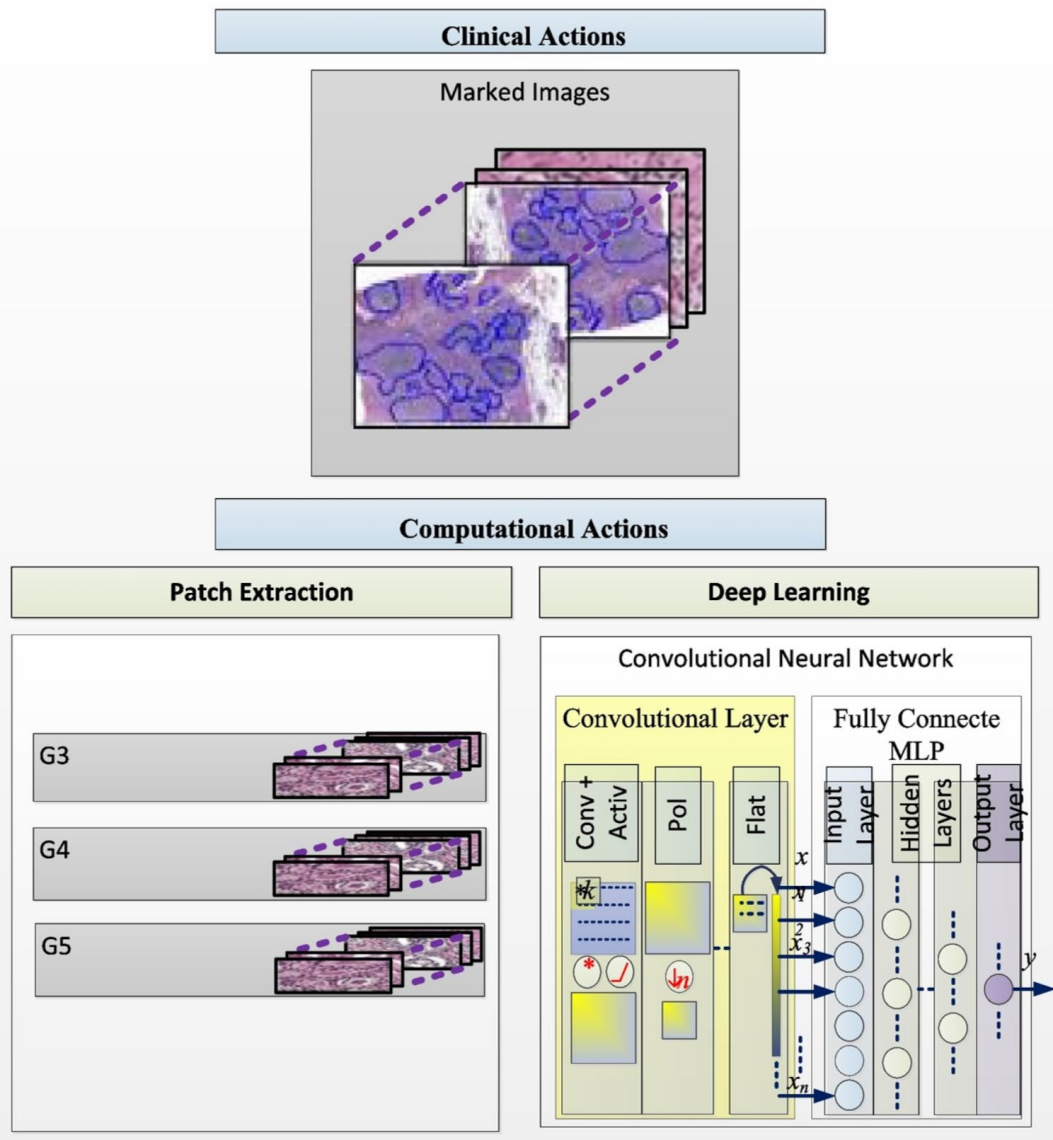
Artificial intelligence approach for detection and grading of prostate cancer in human prostatectomy specimens. © Kudo MS, et al., 2022. Reproduced with permission by the Creative Commons Attribution 4.0 International License, from: Kudo MS, et al., The value of artificial intelligence for detection and grading of prostate cancer in human prostatectomy specimens: a validation study. *Patient Saf. Surg.* 2022, 16:36

**Fig. 2:**
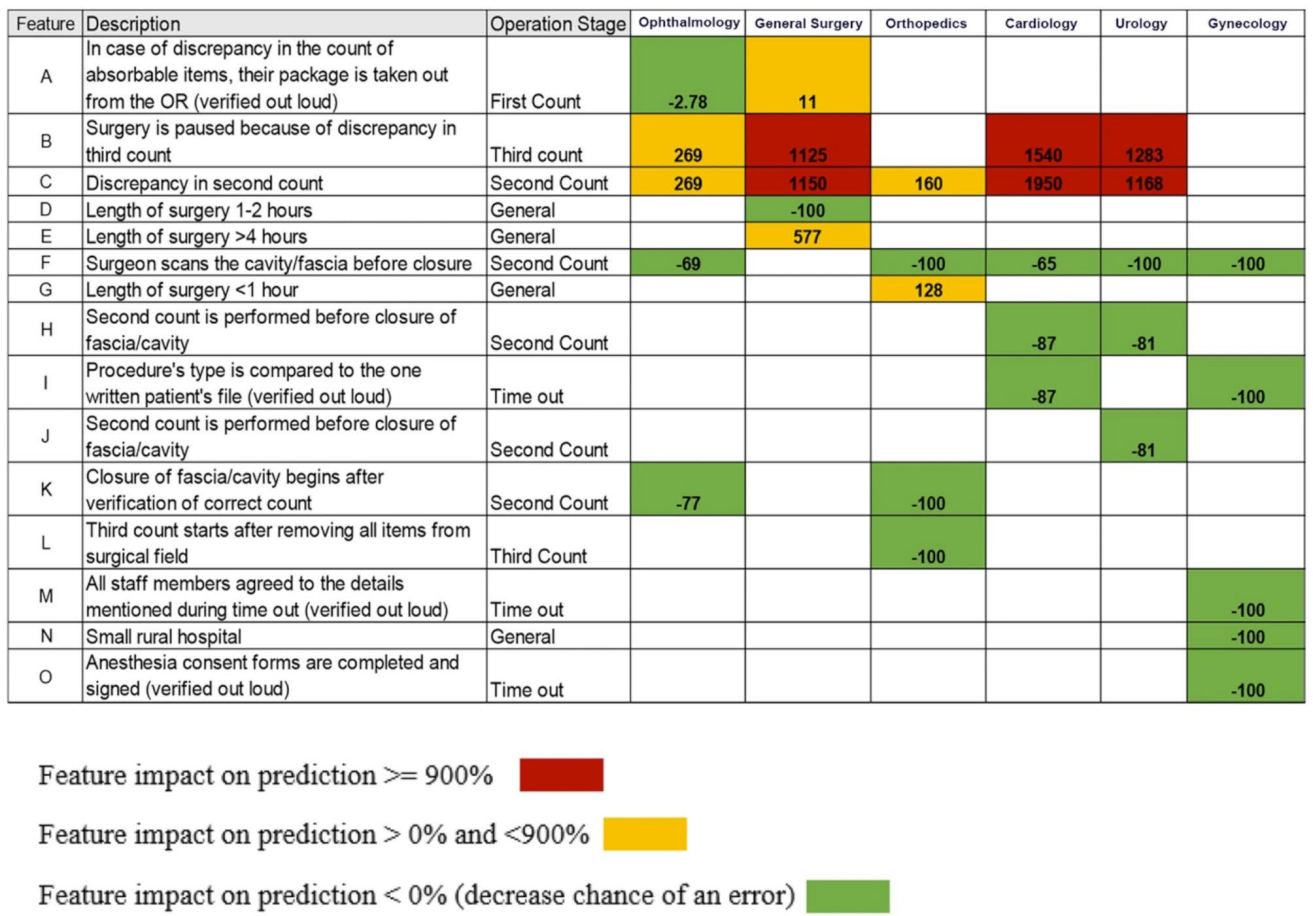
Machine learning approach for prediction of “never events” in the operating room. © Arad D, et al., 2023. Reproduced with permission by the Creative Commons Attribution 4.0 International License, from: Arad D, et al., Factors contributing to preventing operating room “never events”: a machine learning analysis. *Patient Saf. Surg.* 2023, 17:6

**Fig. 3:**
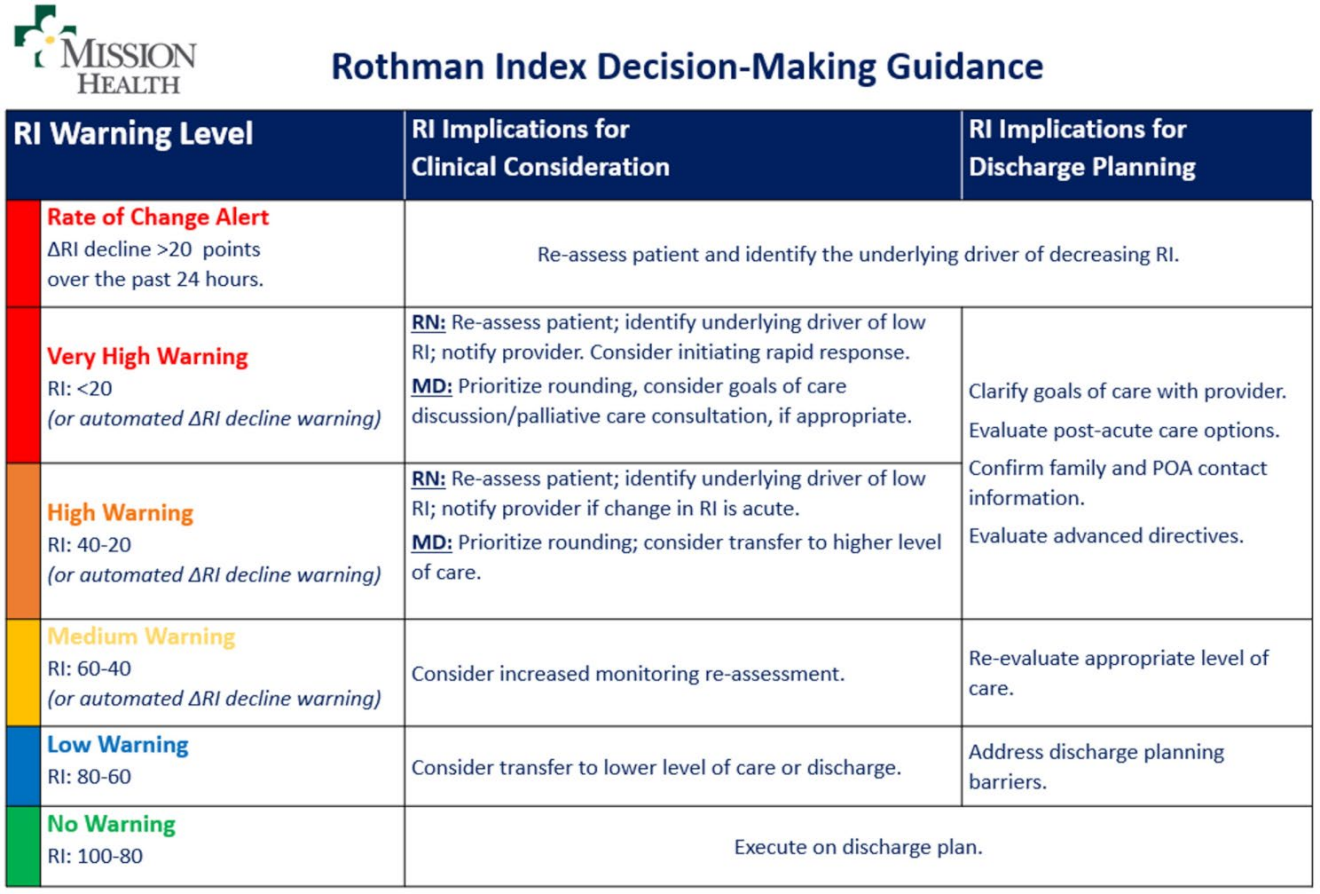
Rothman Index (RI) grading system as a predictive tool for clinical decision-making and patient downgrade/discharge planning, as implemented at Mission Hospital, Asheville, NC. © Stahel PF, et al., 2024. Reproduced with permission by the Creative Commons Attribution 4.0 International License, from: Stahel PF, et al., The Rothman Index predicts unplanned readmissions to intensive care associated with increased mortality and hospital length of stay: a propensity-matched cohort study. *Patient Saf. Surg.* 2024, 18:10

A narrative review article from Kermanshah University of Medical Sciences in Iran provided a compelling state-of-the-art overview on our current knowledge and clinical applications of machine learning technology and artificial intelligence for improving patient safety in spine surgery [11]. The authors provided intriguing insights on how predictive analytics can be leveraged to improve surgical outcomes, how spine surgery practices may design personalized care through machine learning approaches, and how perioperative risks may be mitigated through artificial intelligence for patients undergoing spinal procedures [11]. Importantly, the paper also discussed the potential utility for artificial intelligence technologies to optimize the shared decision-making process with surgical patients. The same research group further investigated the predictive capability of machine learning models in determining outcomes and complications in 329 patients undergoing cervical spine decompression and fusion surgery for spondylotic myelopathy [12]. The authors concluded that their machine learning model represented a highly sensitive predictive tool for surgical complications and postoperative outcomes in the vulnerable cohort of patients with cervical myelopathy [12]. With a similar intent, a prospective proof-of-concept pilot study from Goethe University in Frankfurt, Germany, investigated the “Prehab App“ as a novel digital risk calculator to predict patient outcomes after major surgery [13]. The investigator-initiated Protego Maxima Trial showed that risk assessment using the “Prehab App“ represented a sensitive preoperative predictor of surgical complications and postoperative mortality in a cohort of 267 patients who underwent major surgical procedures in visceral, thoracic, vascular, urological, and gynecological surgery [13].

The trauma research group at the University Hospital Zurich, Switzerland, pursued a new innovative approach by combining the knowledge from large data patient registries with machine learning algorithms, with the intent to elevate the synergy of the predictive capabilities of these individual research tools [14]. The authors combined mutliple data platforms from local, regional, and national registries with various artificial intelligence approaches stratified from narrow, to general, to “super“ intelligence, aimed at predicting and improving postsurgical outcomes in trauma patients [14]. Novel software technologies, such as the “TRAUMA Pathway Explorer“ by IBM Watson Health, are furthermore discussed as available solutions for predicting trauma outcomes based on demographic and physiological patient variables (Fig. 4).

**Fig. 4:**
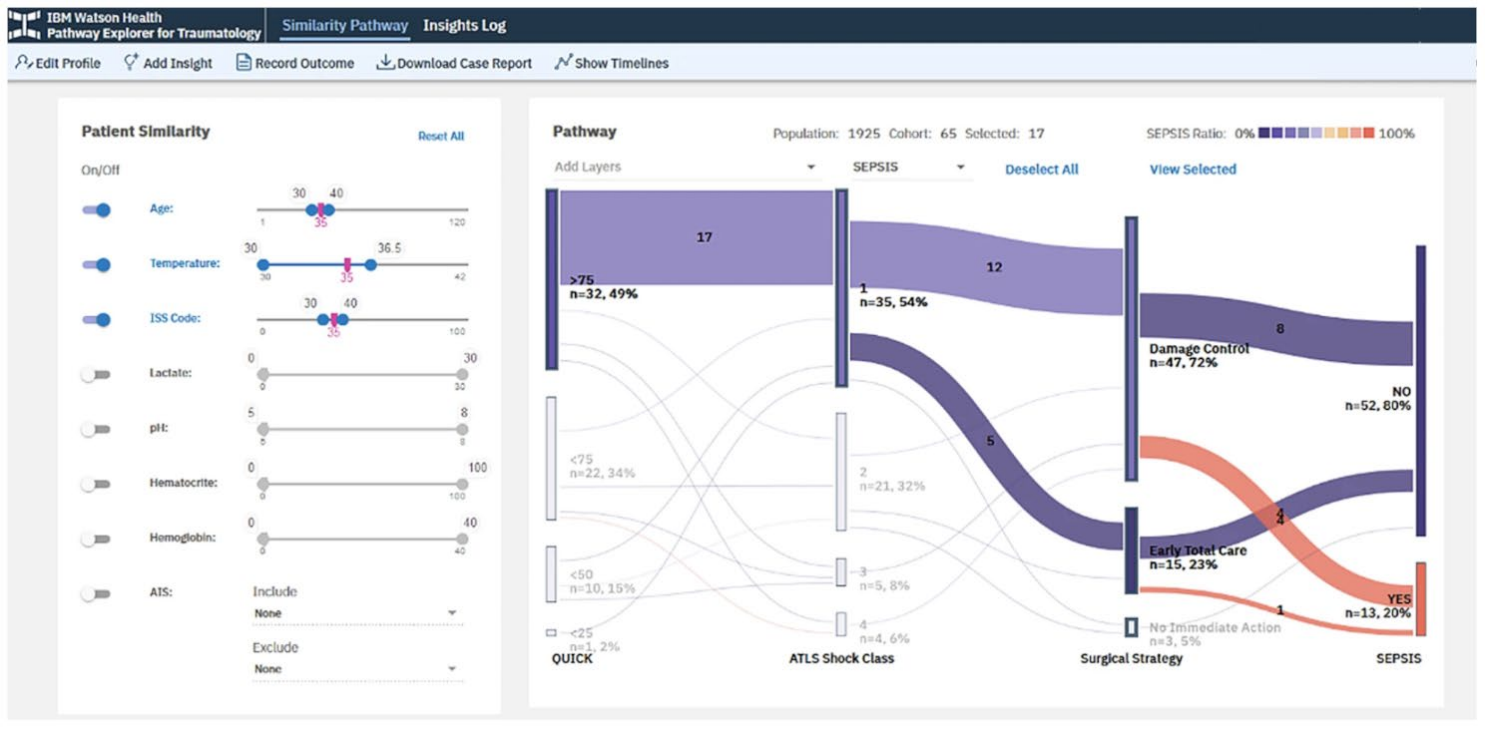
“TRAUMA Pathway Explorer” by IBM Watson Health as an example of a novel software analytics tool designed to predict posttraumatic mortality and patient outcomes. © Pape HC, et al., 2024. Reproduced with permission by the Creative Commons Attribution 4.0 International License, from: Pape HC, et al., The role of big data management, data registries, and machine learning algorithms for optimizing safe definitive surgery in trauma: a review. *Patient Saf. Surg.* 2024, 18:22

The last article in the special collection describes a deep learning-based computer visual model for the automated detection of surgical tools and instruments in the operating room [15]. The authors developed a dataset of based on 1,000 images from more than 13,000 surgical tools to feed a new artificial intelligence model for optimization of the post-surgical instrument count accuracy and prevention of unintentionally retained foreign objects after surgery [15]. The deep learning-based computer vision model demonstrated a high accuracy up to 100% for distinguishing surgical tools from background, and a high sensitivity up to 98.2% for detecting and identifying overlapping instruments (Fig. 5*).* The authors concluded that their innovative deep learning-based visualization model may provide a future artificial intelligence “safeguard“ to prevent surgical “never events” from occurring and harming patients, e.g. by the unintentional retention of foreign objects, such as surgical instruments or lap sponges [16].

**Fig. 5:**
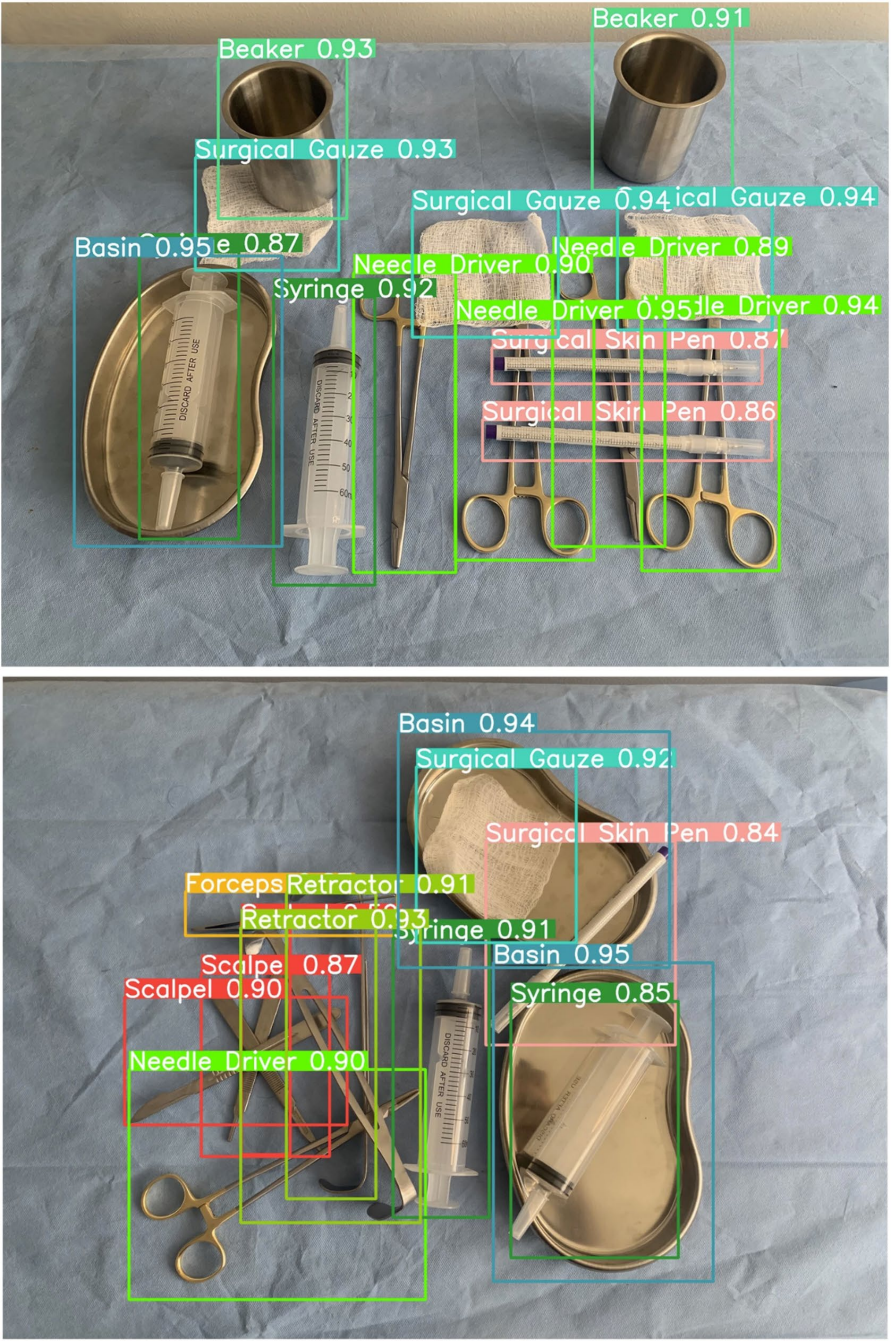
Deep learning-based artificial intelligence computer vision model for automated detection of surgical tools and instruments for improved post-surgery count accuracy and prevention of unintentionally retained foreign bodies. © Deol ES, et al., 2024. Reproduced with permission by the Creative Commons Attribution 4.0 International License, from: Deol ES, et al., Artificial intelligence model for automated surgical instrument detection and counting: an experimental proof-of-concept study. *Patient Saf. Surg.* 2024, 18:24

In summary, the featured articles in the new special collection provide a broad perspective on opportunities for improving patient safety in surgery via modern technologies based on machine learning and artificial intelligence. Ultimately, these novel tools can support, but will not replace, the individual physician’s decision-making process by reducing the margin of human error in judgment and risk of unintentional deviation from established standards of care.

## Data Availability

Please contact the authors for data requests.

## Declarations

### FDA clearance

Not applicable (Editorial).
